# Fast, Efficient Tailoring Growth of Nanocrystalline Diamond Films by Fine-Tuning of Gas-Phase Composition Using Microwave Plasma Chemical Vapor Deposition

**DOI:** 10.3390/ma17122976

**Published:** 2024-06-18

**Authors:** Chunjiu Tang, Antonio J. S. Fernandes, Margarida Facao, Alexandre F. Carvalho, Weixia Chen, Haihong Hou, Florinda M. Costa

**Affiliations:** 1 School of Electronic and Information Engineering, Changshu Institute of Technology, Changshu 215500, China; 2 Department of Physics, I3N (Institute for Nanostructures, Nanomodelling and Nanofabrication), University of Aveiro, 3810-193 Aveiro, Portugal

**Keywords:** nanocrystalline diamond films, nitrogen and oxygen additives, high growth rate, surface roughness, microwave plasma chemical vapor deposition (MPCVD)

## Abstract

Nanocrystalline diamond (NCD) films are attractive for many applications due to their smooth surfaces while holding the properties of diamond. However, their growth rate is generally low using common Ar/CH_4_ with or without H_2_ chemistry and strongly dependent on the overall growth conditions using microwave plasma chemical vapor deposition (MPCVD). In this work, incorporating a small amount of N_2_ and O_2_ additives into CH_4_/H_2_ chemistry offered a much higher growth rate of NCD films, which is promising for some applications. Several novel series of experiments were designed and conducted to tailor the growth features of NCD films by fine-tuning of the gas-phase compositions with different amounts of nitrogen and oxygen addition into CH_4_/H_2_ gas mixtures. The influence of growth parameters, such as the absolute amount and their relative ratios of O_2_ and N_2_ additives; substrate temperature, which was adjusted by two ways and inferred by simulation; and microwave power on NCD formation, was investigated. Short and long deposition runs were carried out to study surface structural evolution with time under identical growth conditions. The morphology, crystalline and optical quality, orientation, and texture of the NCD samples were characterized and analyzed. A variety of NCD films of high average growth rates ranging from 2.1 μm/h up to 6.7 μm/h were successfully achieved by slightly adjusting the O_2_/CH_4_ amounts from 6.25% to 18.75%, while that of N_2_ was kept constant. The results clearly show that the beneficial use of fine-tuning of gas-phase compositions offers a simple and effective way to tailor the growth characteristics and physical properties of NCD films for optimizing the growth conditions to envisage some specific applications.

## 1. Introduction

Recent breakthroughs on material science and nanotechnology such as “stretching diamond to the limit” [1] and the amazing resolution for the major fragile problem of ceramics made of covalent bonds as diamond through “phasing out fracture” [2] stimulate more efforts on the development and research of nanomaterials [3,4]. Among different types of carbon nanostructures [5,6], those produced by various plasma methods, such as graphene [6] and nanocrystalline diamond [7,8], have become the most attractive in recent years. The deposition and development of NCD (of grain sizes usually less than 100 nm) films by CVD methods [7,8,9] is still ongoing because NCD films not only show many characteristics of microcrystalline diamond (MCD, of typical grain sizes larger than 500 nm) films [9], which inherit major advantages of diamond (high hardness, high electron emission efficiency and exceptional chemical inertness [4]), but also exhibit high surface smoothness due to their small grain sizes that potentiate low friction coefficients [4,10,11,12,13]. These unique properties of NCD films extraordinarily enhance the applications of diamond in many new potential fields such as micro- and nano-electromechanical systems (MEMS/NEMS) [12,13] and biomedical applications [13]. Regarding the NCD growth and the reactant gases employed, several different types of methods can be found in the literature. For example, using Ar chemistry in methane with or without hydrogen is a reported way to grow NCD films by various CVD methods [9,14]. This method makes it possible to control the grain size and surface roughness of an NCD film; however, the growth rate is typically lower than 2 μm/h and often less than 1 μm/h [14]. Recently, a work on the deposition of diamond films on Si reported an NCD to MCD transition at very high CH_4_ concentration [15]. The addition of nitrogen into conventional methane/hydrogen gas mixtures has long been explored to deposit CVD diamond for different purposes. These include enhancing the growth rate of a single-crystal diamond [12,16], the formation of a {100}-faceted diamond [10,17,18] and nitrogen-doped diamond [19,20], and the synthesis of NCD films [10,21,22]. Lately, the coupling effects of high methane concentration (7.5–15%) and nitrogen addition level on the morphology and properties of MPCVD diamond films grown on WC-Co substrates by high-power MPCVD system have been studied [10]. In this work, transition from conventional MCD through both the {110} and {100} crystallographic planes of diamond to NCD films occurred upon increasing the amount of N_2_ addition from 0.3 to 0.9 sccm at a microwave power of 3.5 kW and pressure of 20 kPa [10]. Therefore, the impact of nitrogen additive on diamond growth still strongly depends on its content and the other growth parameters, such as methane concentration and microwave power.

Since N_2_ and O_2_ are abundant in nature as main components of air, the addition of N_2_ and O_2_ into reactant gases were both also investigated on the growth of diamond films by hot filament CVD (HFCVD) [23] and MPCVD [24]. Previously, it was found that a variety of diamond films ranging from MCD to NCD can be obtained by only adjusting the relative ratio between N_2_ and O_2_ additives while keeping all the other operating parameters constant [24]. For instance, 0.2 N_2_/0.8 O_2_ addition still resulted in the growth of MCD, while ratios of 0.3/0.7, 0.5/0.5, and 0.8/0.2 resulted in the formation of NCD films [24,25]. This clearly demonstrates the versatility of the approach by N_2_ plus O_2_ addition in high-power MPCVD diamond growth, enabling not only the transition from MCD to NCD but also tuning the quality, morphology, and orientation or texture [24]. Furthermore, since 0.8 N_2_/0.2 O_2_ is close to that of air, 1 sccm air was directly introduced into a 4% CH_4_/H_2_ mixture using different microwave powers up to 4.0 kW [11,26,27], resulting in NCD films. In fact, a lower amount of air addition (0.5 sccm) into 5% CH_4_ in H_2_ plasma resulted in the formation of diamond films with {100} crystallographic planes grown on pure Co substrate at microwave power of 2.4 kW using the same reactor [28]. However, so far, the amount of both N_2_ and O_2_ addition including the special case of air into CH_4_/H_2_ plasma at high-power conditions has been limited to 1 sccm or less, as summarized in Table 1 for clarity. Hence, regarding the effect of both N_2_ and O_2_ addition on NCD growth, questions arise. For example, can NCD films be formed with higher amounts of oxygen and nitrogen additives under different conditions? How will the characteristics of NCD films, such as grain size and surface roughness, be affected by both the amounts of N_2_ and O_2_ additions and the other deposition parameters? Accordingly, further investigation on the proper range of nitrogen and oxygen addition on NCD growth are mandatory for optimization of the growth parameters for some specific applications. 

To answer the above-mentioned questions and extend the possible parameter ranges for growth of NCD films, in this study, we designed and explored new series of experiments by employing N_2_ flow fixed at 1 sccm and oxygen additions ranging from 1 to 3 sccm into 4% CH_4_/H_2_ mixtures. For this set of growth runs, high microwave power ranging from 3.2 to 3.5 kW was used under several growth conditions (namely by varying substrate surface temperature and the deposition time), successfully fabricating NCD films attaining high growth rates up to 6.7 μm/h.

Exploitation of this new growth parameter range offers a simple way to tailor the growth of large area uniform NCD films at high growth rate toward industrial level applications upon proper adaptation of standard high power MPCVD approach. 

## 2. Materials and Methods

### 2.1. Design and Deposition of Diamond Films

For this study, three series of diamond depositions were designed and conducted with different amounts of N_2_ and O_2_ additives under various conditions using a 5 kW MPCVD reactor (PDS-18, ASTeX Inc. Woburn, MA, USA). The pressure was kept at 105 Torr, and the CH_4_/H_2_ ratio was fixed at 4% for all the samples, whereas the absolute gas flow was 16/400 sccm (DNO1, DNO2, DN3, and DNO5–DNO7) and 20/500 sccm (D4 and DNO8–DNO10). All the <100>-oriented single-crystal Si substrates were pre-scratched with a diamond powder with a size of 0~0.5 μm to enhance diamond nucleation. The denoted sample name and the other corresponding growth parameters, such as microwave power, substrate thickness, amount of nitrogen and oxygen, and deposition time, are listed in Table 2, Table 3, and Table 4, respectively. Two molybdenum holders were used, both having 2 inches in diameter but different thermal contact areas to the water-cooled steel base. They are denoted as type 1, which was used for the growth of the first nine samples, and type 2, which was only applied to the last sample DNO10. When a type 2 holder (with much less thermal contact area relative to the support beneath) was used, a much higher surface temperature of the Si substrate could be obtained under the same growth conditions. During the deposition process, the temperature of water-cooled dish right beneath the holder was measured by an in situ thermocouple and referred in the tables as the Mo holder temperature.

Sample DNO1, a highly <100>-oriented NCD film achieved on 3 mm-thick, 2-inch Ø Si wafer using a small, equal amount (0.5 sccm) of N_2_ and O_2_ addition, served as starting point [29]. Stimulated by this previous work, a new set of experiments was designed by increasing the amount of both N_2_ and O_2_ additives to 1 sccm each using smaller substrates (1 × 1 cm^2^, 1 mm-thick, <100> oriented silicon), hereafter termed DNO2. Notably, NCD films of very high growth rates up to 9.6 μm/h had been obtained with pure N_2_ addition (1.0 and 1.6 sccm) at a high power from 3.0 up to 4.0 kW on similar 1 mm-thick Si slices [21]. The sample grown with only N_2_ additive at a power of 3.5 kW was denoted as DN3, while D4 was a conventional MCD sample grown with pure CH_4_/H_2_ mixture under similar conditions. Some of the other growth parameters of this first set of samples are given in Table 2. 

Substrate temperature is one important growth parameter among others such as power and pressure. In the high-power MPCVD system used in the present work, substrate was heated by the hot plasma and cooled by the stage that supports the holder. Therefore, temperature was not a direct operable parameter, being mainly controlled by microwave power and pressure and the type of Mo holder (type 1 or 2). When the above conditions are kept constant, substrate surface temperature can be varied by substrate thickness, with thicker substrates leading to higher surface temperatures. To systematically check substrate temperature effects on NCD growth under the new conditions employed here for DNO2, a substrate batch of <100>-oriented, single-crystal Si wafers of 2 inches in diameter and different thickness ranging from 1 mm to 5 mm was selected for the second series of deposition runs. The growth parameters are listed in Table 3.

Inspired by these new results, the third series of deposition runs were designed and carried out by further increasing the amount of O_2_ additive, while keeping that of N_2_ fixed at 1 sccm. As previously reported, when the amount of oxygen addition was four times that of nitrogen (namely O_2_/N_2_ was 0.8/0.2 sccm), the diamond product was still MCD [24], while the critical change for NCD formation occurred at O_2_/N_2_ = 0.7/0.3 sccm [25]. Therefore, a maximum of three times of O_2_ addition as that of N_2_ was chosen for the third series of experiments, with short and long deposition time, respectively, to study the evolution of film structure, grain size, and surface roughness with film thickness. Both types of Mo holder were also used to further check temperature effects on diamond growth under the new growth conditions. The third set of samples were grown on silicon wafers of 2 inch in diameter and 3 mm in thickness, listed in Table 4 for comparison.

### 2.2. Adjustment and Simulation of Substrate Surface Temperature

As a summary, for this work, the Si substrate surface temperature was adjusted by three following means: (i) slightly varying microwave power (Table 2), (ii) using Si substrates of different thickness (Table 3), and (iii) changing the type of Mo holder (Table 4). For instance, in the case of the first set of diamond deposition on small, 1 mm-thick silicon slices, the difference in the Mo holder temperature was about 60 °C between sample DNO2 and D4, which resulted from the 300 W lower power used for sample D4. Please note the fact that the reactor configuration did not allow for a direct measurement of the substrate surface temperature by pyrometry due to the location of the view port (the plasma stands in front of the substrate surface line of sight). To evaluate and compare the effect of substrate surface temperature on NCD growth under the conditions employed here, we ran a simulation to find out the temperature distributions of Mo holder and Si substrates using COMSOL Multiphysics 5.3a and the Heat Transfer module. In the simulations, we assumed that the temperature of the bottom surface of the holder was the same as the temperature measured by the thermocouple located just below the holder, in the cooled dish. The heat in flow from the plasma was assumed to be homogeneous all over the free surfaces of Si and Mo holder and adjusted around 5 × 10^6^ W/m^2^ to achieve the Si top surface temperatures in the range from 700 up to 900 °C suitable for diamond growth under similar conditions. 

### 2.3. Characterization of Diamond Films 

Scanning Electron Microscopy (Hitachi S-4100 and SU-70, Tokyo, Japan), operated at 25 kV, and micro-Raman spectrometry (Horiba HR800, Kyoto, Japan) operated at a laser wavelength of 442 nm) were used for morphological and structural characterization of the diamond samples, respectively. The average growth rate of the samples was obtained by measuring the film thickness using SEM cross-sectional image first and then divided by the deposition time. The raw Raman spectra underwent subtraction of luminescence background and normalized according to the highest peak in each spectrum for clarity of presentation and comparison. X-ray diffraction (XRD) characterization was also performed in a high-resolution 4-circle mode using a diffractometer X’Pert PRO MRD (PANalytical, Almelo, The Netherlands) using Cu radiation. Powder diffraction patterns (conventional θ-2θ scans) were recorded in reflection geometry and using high intensity X-ray optics. Surface roughness of the diamond samples was measured using a NANOVEA ST400 optical profilometer (Irvine, USA).

## 3. Results

### 3.1. Growth of NCD Films Using Increased Equal Amount of N_2_ and O_2_ Additives

**Figure 1:**
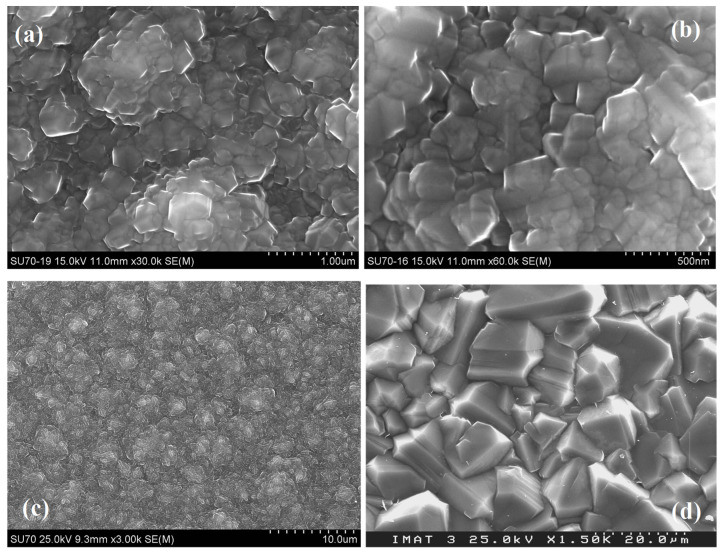
SEM micrographs taken from the top growth surface of the first set of diamond samples (**a**,**b**) DNO2 at a microwave power of 3.5 kW with an equal amount of N_2_ and O_2_ additions, (**c**) DN3 with N_2_ addition only, and (**d**) D4 using only CH_4_/H_2_ mixture.

To identify the role of N_2_ and O_2_ addition on NCD growth, the new sample DNO2 was grown with higher equal amount (1 sccm) of N_2_ and O_2_ addition and analyzed in detail and compared with the other two samples grown under similar base conditions, namely DN3 (higher amount of N_2_) and D4 (CH_4_/H_2_ mixture only). Morphological characterization by SEM, structural assessment by Raman spectroscopy, and grain size and orientation or texture analysis by XRD were undertaken. Figure 1 shows the SEM images taken from the growth surfaces of the above-mentioned samples. Different morphologies among the samples can be seen clearly. Sample DNO2 (Figure 1a) was composed of clusters of irregular shapes from 1 to 10 μm in size. The higher-magnified SEM micrograph (Figure 1b) illustrates that the big clusters were composed of fine grains whose size was in the range of several tens up to 100 nm, and some fine grains faceted with (100) planes or (111) planes, indicating better crystallinity. This morphology is distinct from the cauliflower-like morphology of NCD film as that of sample DN3 grown with N_2_ additive only, as shown in Figure 1c. Moreover, it is also different from the cauliflower-like morphology of NCD films grown with Ar chemistry reported in the literature [30]. In contrast, film D4 was constituted by smooth well-defined polyhedral crystallites with sharp edges of different sizes ranging from around 1 μm up to 20 μm as shown in Figure 1d, a typical common feature of high-quality polycrystalline CVD diamond films grown with CH_4_/H_2_ plasmas [24]. This result indicates that new sample DNO2 indeed possesses nanocrystalline nature in accordance with its general appearance.

**Figure 2:**
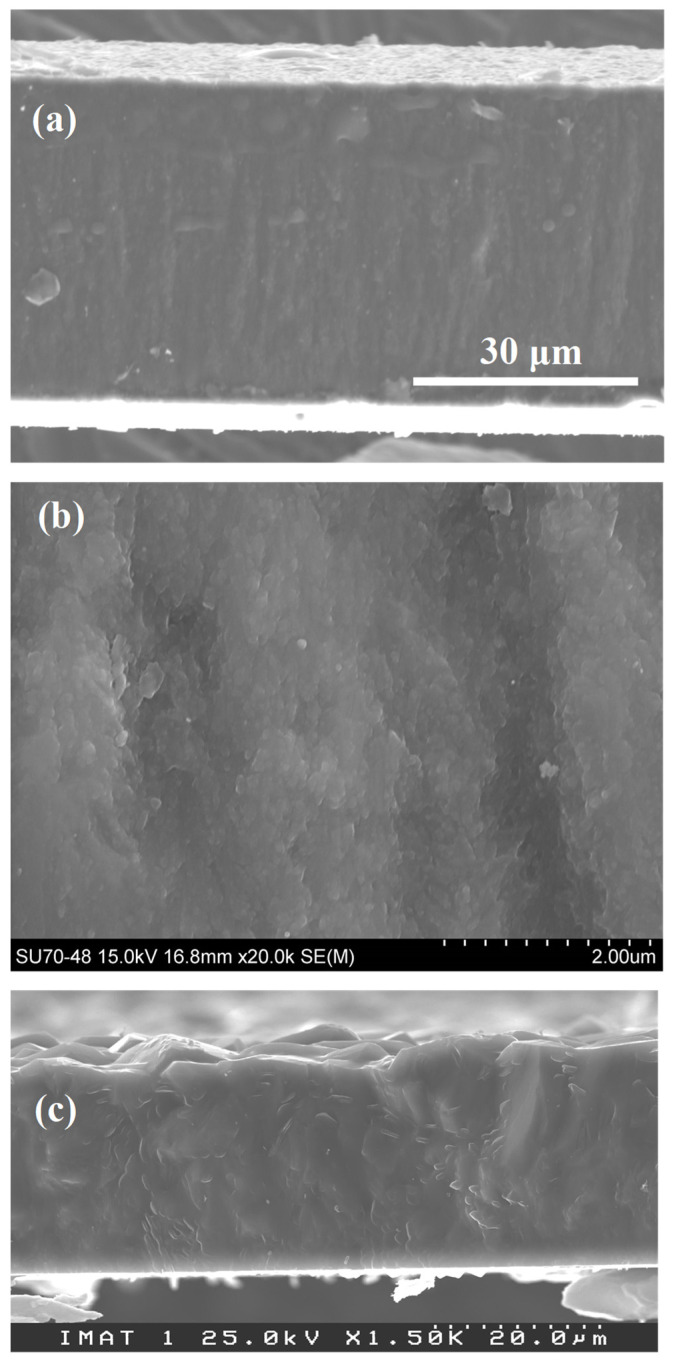
Cross-sectional SEM micrographs of the free-standing diamond films: (**a**,**b**) DNO2 and (**c**) D4 grown on a 1 mm-thick, 1 × 1 cm^2^ Si slice with an equal amount of N_2_ and O_2_ addition at a power of 3.5 kW.

For further comparison of the nanocrystalline nature of the grains in sample DNO2 with the microcrystalline ones in D4, grown under similar base condition (namely without N_2_ nor O_2_), full cross-sectional views of both films are shown in Figure 2. One can see from Figure 2a that the grain size and surface roughness of sample DNO2 did not change obviously with the film thickness (or deposition time), a common feature of NCD films [25]. The cross-section of DNO2 sample was magnified (Figure 2b) to clearly depict the nanocrystalline nature of the grains. In contrast, the D4 sample (Figure 2c) exhibited a typical structure of microcrystalline film, namely the grain size and surface roughness increase with film thickness (or deposition time), a characteristic of large-grained polycrystalline diamond film, which hinders direct applications in fields such as coatings for wear-resistant parts [10]. From the full cross-sectional SEM micrographs, the thickness was measured and thus the samples’ average growth rate could be calculated. DNO2 film showed a 35.2 μm thickness and thus an average growth rate of 6.7 μm/h, while for film D4, a thickness in the range from 23.8 μm to 27.6 μm indicated an average growth rate up to 2.5 μm/h, as listed in Table 2. Hence, one can conclude that the simultaneous addition of N_2_ and O_2_ enabled a more than two-fold increase of the deposition rate when comparing samples DNO2 and D4.

In Table 2, the average growth rates for all samples are listed. For instance, compared with DNO1, the NCD film previously grown at 3.0 kW with 0.5 sccm N_2_ and 0.5 sccm O_2_ [29], the average growth rate of sample DNO2 was more than double that of DNO1 and was comparable to DN3 sample grown with only N_2_ (1.6 sccm). A previous study has shown that with 1 sccm air addition, the highest growth rate achieved for NCD films was 3.4 μm/h at a microwave power of 3.2 kW [26] and further increased to 4.6 μm/h when the microwave power rose to 4.0 kW [27]. In different growth chemistries, namely those using inert gas Ar/CH_4_ developed to produce NCD, often resulting in grain sizes down to 10 nanometers, the typical growth rates are lower than 1 μm/h, with the highest reported attaining 1.7 μm/h [14]. Therefore, concerning the growth rate yield, a higher amount of N_2_ and O_2_ offers a fast way to fabricate NCD films by high-power MPCVD. 

**Figure 3:**
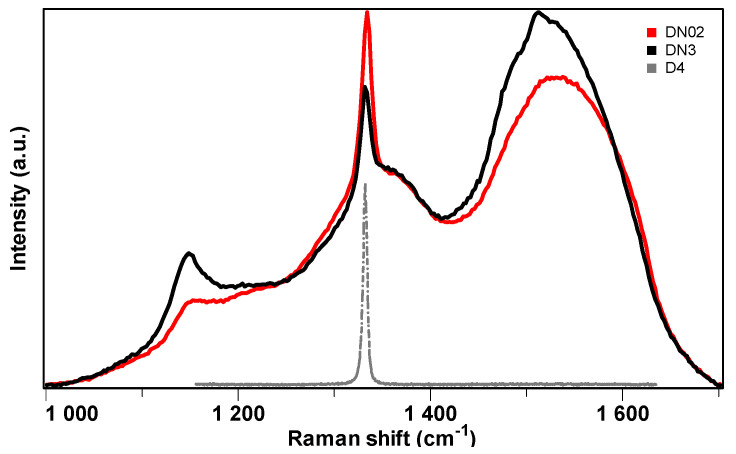
Visible Raman spectra taken from the center of the growth surface of the NCD films DNO2 and DN3 and the transparent MCD film D4 for comparison.

To identify the diamond nature of the as-grown new sample DNO2, Figure 3 shows its micro-Raman spectrum taken from the center of the film along with that of DN3 for comparison. The appearance of sharp peak around 1333 cm^−1^ in the Raman spectra clearly confirms the diamond nature of both films. However, the diamond peak of the sample DNO2 was much stronger and narrower than that of DN3 film, denoting the better diamond crystallinity of the DNO2 sample. Other features also appeared in the Raman spectra, namely a hump at ~1140 cm^−1^ and a broad band centered around 1500 cm^−1^, composed of two sub-bands at ~1475 cm^−1^ and ~1550 cm^−1^. The former can be assigned to trans-polyacetylene (1140 cm^−1^ and 1475 cm^−1^), typically present at the grain boundaries of NCD films, while that at ~1550 cm^−1^ was due to the sp^2^-related G band [31]. Opposite to the stronger diamond peak, these features were much weaker in sample DNO2 than in sample DN3, indicating less non-diamond component in sample DNO2. This result was also compatible with the faceted crystalline structure of DNO2 sample seen in SEM images (Figure 1a,b) and was due to the addition of O_2_. In contrast, the Raman spectrum of D4 film only showed a strong sharp diamond peak centered at around 1333 cm^−1^ with a linear background. The full width at half maximum (FWHM) of the diamond peak in this sample was 3.5 cm^−1^, close to that of 2.6 cm^−1^ for the IIa natural single-crystal diamond used for calibration. This clearly evidences the higher crystalline quality of D4 film, which is consistent with its smooth faceted crystallite morphology, as shown in Figure 1d.

**Figure 4:**
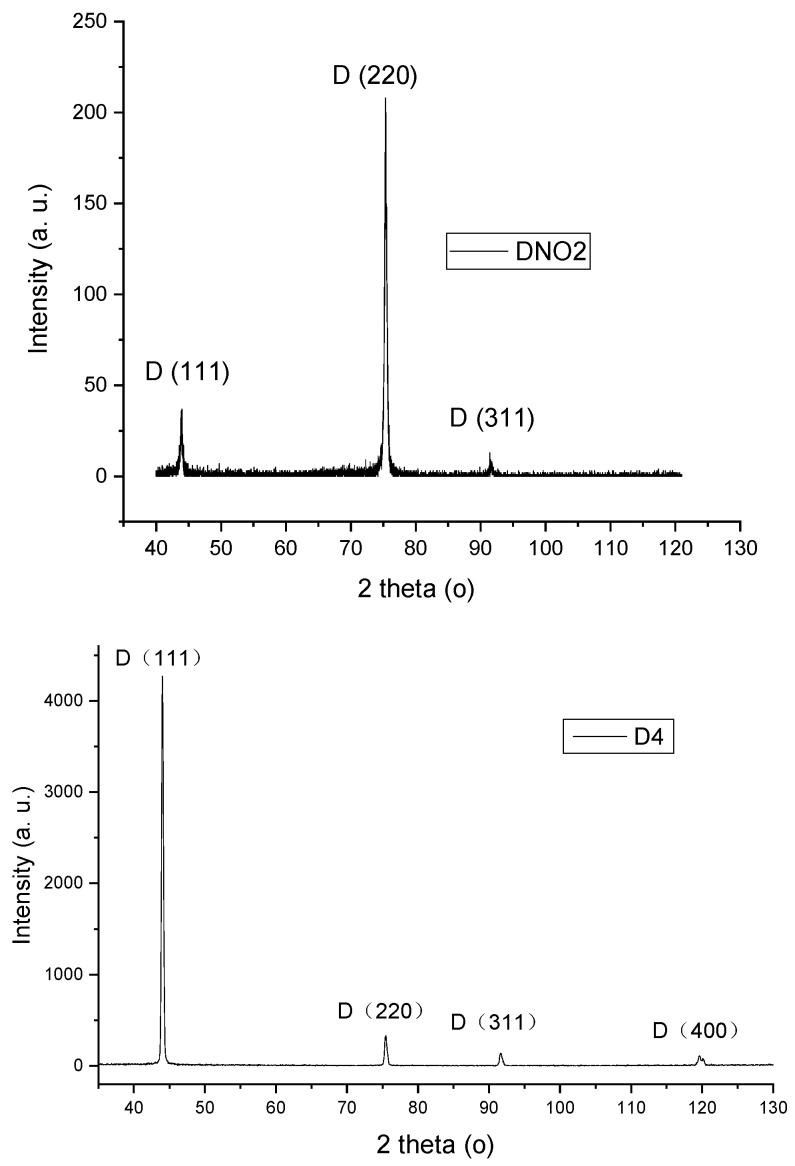
Conventional θ-2θ scans by XRD of representative NCD film DNO2 grown with equal amount of N_2_ and O_2_ addition and D4 produced under the base conditions for comparison.

The samples’ diamond crystalline nature was also corroborated by their XRD patterns, as shown in Figure 4. Additionally, this technique allowed us to estimate the average grain size from the FWHM of the strongest diamond diffraction peak in the case of NCD films. Figure 4 shows the conventional XRD patterns (θ/2θ scans) of NCD DNO2 and MCD D4 films for comparison. For sample DNO2, the diamond (220) diffraction peak at 2θ = 75.3° was the strongest among other peaks, such as the (111) peak at 2θ = 43.9° and (311) peak at 2θ = 93.1°. Its intensity was 5.5 times higher than that of the (111) peak, very much exceeding 0.25, typical of standard randomly oriented diamond power. This indicates a <110> preferential orientation, a common feature for most NCD and MCD films [11,24,32]. According to the FWHM of the dominating (220) diamond diffraction peak, and using the well-known Sherrer’s equation [33], the average grain size of the fine grains comprised by the film was calculated to be about 31 ± 10 nm. It is worth mentioning that the average grain size of sample DN3 (1.6 sccm N_2_, same base conditions) was 38 ± 10 nm, calculated from the FWHM of the strongest <220> peak [21], while that of DNO1 film previously grown with 0.5 sccm N_2_ and 0.5 sccm O_2_ at 3.0 kW, with a strong [100] preferential texture, was 60 ± 10 nm upon the analysis of the breath of the strongest (400) diffraction peak [29]. Therefore, XRD analysis (Figure 4) not only confirms the crystalline diamond nature of sample DNO2 but also evidences its nanocrystalline nature. On the other hand, the XRD pattern of the MCD film D4 grown without N_2_ nor O_2_ showed that the diamond (111) diffraction peak at 43.9° was the strongest one and dominated the pattern, while the other diamond diffraction peaks were rather weak, such as (220) at 2θ = 75.3°, (311) at 2θ = 93.1°, and (400) at 2θ = 119.5°. The intensity ratio between the (111) and (220) peaks of sample D4 was 12.9, higher than the typical 4.0 for a standard randomly oriented diamond powder, indicating a slight <111> preferred orientation. This corroborates with its general morphology consisting of crystallites composed of {111} faceted pyramids as shown in Figure 1d. 

Therefore, as a summary of the first attempt of novel experiments, one can conclude that an NCD film of <110> preferred orientation with even smaller grain size was achieved with a much higher growth rate by raising the amount of N_2_ and O_2_ additives under conventional base conditions for growth of high-quality polycrystalline diamond films. It is worth noting that NCD films grown with air addition into 4% CH_4_/H_2_ plasma under identical conditions at power 3 kW by MPCVD showed increased intensity ratio between the <220> and <111> diffraction peak of diamond with deposition time from 20 min up to 14 h [11]. This indicates that <110> preferential orientation is indeed a common feature of NCD films [11,32].

**Figure 5:**
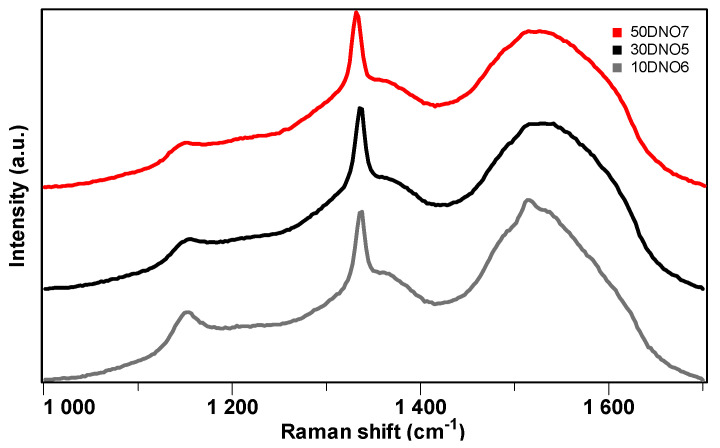
Visible Raman spectra taken from the center of the growth surface of samples DNO5–DNO7, grown with equal amount of N_2_ and O_2_ addition at power 3.5 kW on 2-inchSi wafers with thicknesses of 1 mm, 3 mm, and 5 mm, respectively.

To check the temperature range suitable for NCD formation with an equal amount of N_2_ and O_2_ additives under the new condition employed for sample DNO2, the second series of experiments was designed and conducted on 2-inch Ø Si wafers of various thickness ranging from 1 mm to 5 mm. During the deposition process of this second set of samples, the water-cooled dish temperatures measured by the in situ thermocouple were very close, around 350 °C (Table 3), indicating the operating stability of the reactor. Under the conditions employed here, a thicker Si substrate leads to higher substrate surface temperature. This way, the Si substrate surface temperature where diamond growth takes place can be systematically varied while keeping all the other process parameters fixed. Data regarding this set of samples are given in Table 3. A naked eye inspection of the film surface appearance allowed us to observe that the growth surfaces of these samples were relatively uniform and smooth in comparison with large-grained polycrystalline diamond films. To identify the diamond nature of these new samples, their micro-Raman spectra of the second series of samples DNO5–DNO7 taken from the center of the samples were collected and are illustrated in Figure 5. After removing the luminescent background, three major common features could be clearly identified among these three new samples. Firstly, a prominent sharp peak appeared at around 1333 cm^−1^, indicating the diamond nature of all the samples. Secondly, a strong broad band was evident centered around 1550 cm^−1^, which came mainly from non-diamond component, namely the sp^2^-related G band (~1550 cm^−1^) [31]. Thirdly, a trans-polyacetylene related weaker peak arose at ~1140 cm^−1^ (usually along with a band at 1480 cm^−1^), typically present at the grain boundaries of NCD films [31]. The three Raman spectra of the samples look similar, indicating their analogous structural nature within the temperature range investigated. These results clearly demonstrate that this set of growth conditions is suitable for NCD deposition.

Surface roughness measurements of this set of NCD samples, as listed in Table 3, show that the root mean square (RMS) roughness was in the range of 291–397 nm, slightly increasing with decreasing Si substrate thickness from 5 mm to 1 mm, i.e., with decreasing substrate surface temperature. In contrast, the RMS roughness of the MCD film D4 was about 2 μm. These results clearly demonstrate that new NCD films obtained here had low surface roughness in accordance with their fine grain sizes. 

Modelling simulation makes it possible to infer on a temperature difference between the surface of each Si substrate (from 1 mm to 5 mm thick), a way to overcome the difficulty of directly measuring the substrate surface temperature during deposition due to the opaque hot plasma and the window design of the MPCVD system. For diamond growth by high-power MPCVD under similar power conditions, usually the deposition temperature is reported to be around 700–1000 °C [10,34,35,36]. This can serve as a reference for the simulation of the surface temperatures of Si substrates used for NCD deposition here. For example, assuming a homogeneous heat flow, the simulated surface temperatures at the center of each Si wafer span from around 672 °C for 1 mm-thick Si, around 764 °C for 3 mm-thick Si, and up to 864 °C for a 5 mm-thick Si wafer, respectively, in agreement with the successful deposition of NCD films. In other words, a surface temperature difference of about 200 °C can be obtained using Si wafers with a thickness difference of 4 mm under the same growth conditions. 

From Table 3, one can see that after 2 h deposition under identical conditions, the average growth rate was 3.5 μm/h for sample DNO6 grown at the lowest substrate temperature, 4.2 μm/h for sample DNO5 grown at the middle temperature, and 3.8 μm/h for sample DNO7 grown at the highest temperature. They were higher than 2.6 μm/h, the average growth rate of sample DNO1. This indicates the efficiency of this approach to enhance the growth rate of NCD films by raising the amount of N_2_ and O_2_ additives. 

### 3.2. Formation of NCD Films Using Higher Amount of O_2_ Than N_2_ Addition

**Figure 6:**
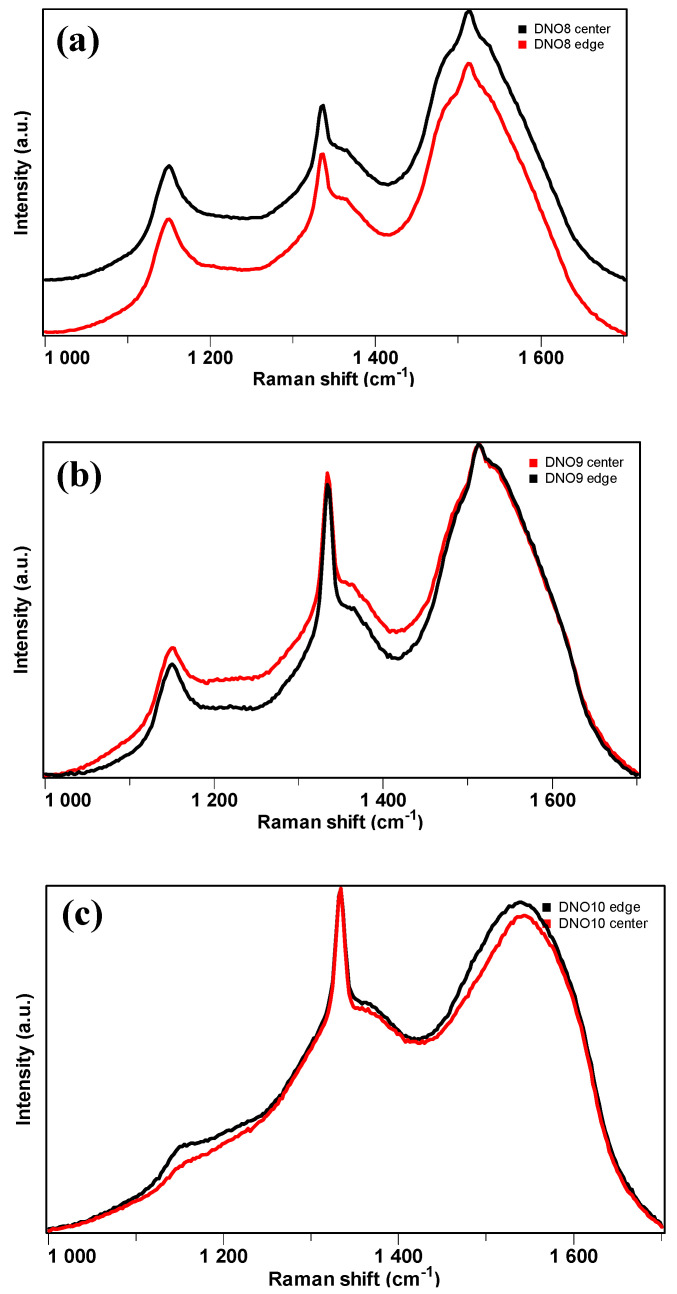
Micro-Raman spectra taken from the center and edge of the growth surface of the samples grown on 3 mm-thick Si wafers: (**a**) DNO8, (**b**) DNO9, and (**c**) DNO10.

To find out more absolute amount and relative ratios between O_2_ and N_2_ additives suitable for NCD growth, the third series of samples were produced using three times more O_2_ than N_2_ additive. To study the surface structural evolution with time and film thickness, the duration of deposition run was set to be 40 min as a “short” run for a thin film and 20 h as a “long” growth for a thick film. An early study of NCD growth with air addition into 4% CH_4_/H_2_ plasma at 3.0 kW and 80 Torr from 2.5 min up to 14 h deposition under identical growth conditions demonstrated that continuous NCD films were only obtained with a sufficient growth time of longer than 20 min [11]. To explore more different conditions, a higher top surface temperature of Si substrate was also tested by using the Mo holder 2, using the same base conditions as that for film D4 as listed in Table 4. Micro-Raman spectra taken from both the center and edge of these samples, namely from DNO8 to DNO10, are shown in Figure 6, to identify their diamond nature and further check the uniformity of the diamond growth. The sharp peak around 1333 cm^−1^ was visible in all the Raman spectra of the samples, clearly indicating their diamond nature. Other features came from TPA at ~1140 cm^−1^ and a non-diamond component, a broad band centered around 1500 cm^−1^. These other features have been described in detail for Figure 3. First, the two Raman spectra taken from the center and edge of the same sample were very similar. This indicates that the diamond growth was uniform on the big Si wafers in terms of crystalline quality. When we compare the difference among the samples, a significant decrease in intensity of the band around 1140 cm^−1^ from sample DNO8 to DNO9 and DNO10 can be observed. This means that the content of TPA decreasing with deposition time was noticeable. Comparing the TPA peak between samples DNO9 and DNO10, one can see that with the increasing substrate temperature, the TPA peak became very weak, in accordance with the properties of TPA [31].

**Figure 7:**
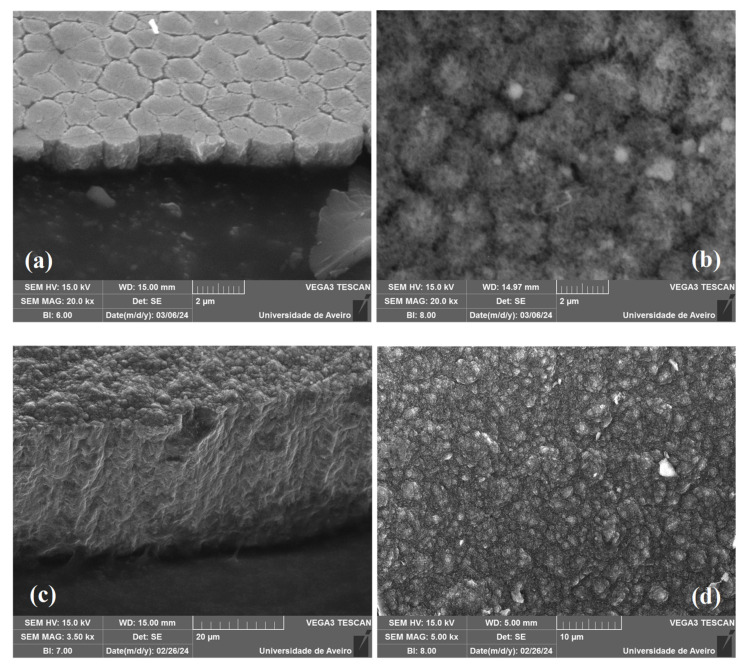
SEM micrographs taken from the cross-section tilted at a 45-degree angle (**a**); the top growth surface (**b**) of the thin sample of DNO8 after 40 min of deposition; from the cross-section tilted at a 45-degree angle; (**c**) and the top growth surface (**d**) of the thick film DNO9 after 20 h of growth fabricated with 3 (sccm) O_2_ and 1 (sccm) N_2_ addition at a power 3.2 kW.

Comparing samples DNO8 and DNO9, one can appraise surface structural evolution with the deposition time and film thickness under identical growth conditions. Their SEM micrographs taken from the full cross-section for thickness measurement tilted at 45 degrees and their top growth surface are shown in Figure 7. From Figure 7, one can see that the grain size and surface roughness of the growth surface did not increase significantly with the film thickness and deposition time, in accordance with the general feature of NCD films reported in the literature [11,25], while for MCD films, the grain sizes increase with growth time due to columnar growth [37]. From Table 4, one can see that the same growth rate was obtained indicating the reproducibility of the NCD film growth. RMS surface roughness increases only slightly from 306 nm to 524 nm after 20 h of growth. Comparing samples DNO9 and DNO10, the major difference on growth parameter was the substrate temperature modified using different types of Mo holders with “large” or “small” contact areas with the water-cooled dish underneath. Using the same operating parameters, the surface temperature difference on the top center of the two Si substrates was about 350 °C as obtained by simulation. Comparing samples DNO10 with DNO9, higher substrate temperature resulted in a larger growth rate of 3.8 μm/h, but the RMS surface roughness slightly decreased to 466 nm, with the latter result being consistent with results obtained in the second run at which the roughness also decreased with increasing temperature. Therefore, one can conclude that NCD films were successfully achieved with N_2_ (1 sccm) and O_2_ (3 sccm) additives at a power of 3.2 kW under the base conditions used for D4, but also for the longer time and higher substrate temperature. These results further extend the parameter range of N_2_ and O_2_ addition for NCD formation using a high-power MPCVD.

As mentioned before, the substrate temperature can be modified by microwave power, thickness of the substrate, and the design of the Mo holder in a high-power MPCVD system. The present results, namely the difference of about 200 °C, resulted from the 4 mm discrepancy in Si thickness (second set of deposition runs) and about a 350 °C higher difference in the top surface temperature of Si substrates, which were 3 mm thick when using a Mo holder 2 of less contacting area compared to a holder 1 of more contacting area (third series of growth), further evidencing the validity of the approach to tune temperature and the wide temperature range for the formation of NCD films with different amounts of N_2_ and O_2_ additives under base conditions, for instance, as those of D4.

## 4. Discussion

### 4.1. New Operating Parameter Range for the Formation of NCD Films with Different Amounts of O_2_ and N_2_ Addition

Combining the experiments conducted here and carried out before, the results are discussed as follows. First, NCD films with a high growth rate and various characteristics can be fabricated by adjusting only the amount of N_2_ and O_2_ additives while keeping all the other growth parameters fixed. Second, using a 5 kW MPCVD system, an extended possible parameter range for the formation of NCD films can be envisaged under base conditions (4% CH_4_/H_2_ mixtures, 95–110 Torr pressure, 2.0–4.0 kW microwave power, N_2_ (0.5–1.6 sccm), and O_2_ (0 to 3 sccm), as exposed in Table 5.

From Table 5, one can see that the absolute amount and relative flow ratios between N_2_ and O_2_ additives have significant influence on the diamond product (MCD or NCD). Based on the present and previous work [24], one can deduce that for the formation of NCD films, the amount of O_2_ can be any value in the range from zero up to three times that of N_2_, when the amount of N_2_ is fixed at 1 sccm, under base conditions similar or equal to that for D4. Therefore, provided that N_2_ is present to a certain threshold concentration, when the amount of O_2_ is two times that of N_2_, the deposit will certainly be NCD, a relevant guide for designing future experimental work on the optimization of NCD growth conditions. Therefore, the increased amounts of N_2_ and O_2_ additives with different ratios for the formation of NCD films studied here not only extend the parameter range for NCD growth but also evidence the stability of the operating system we have been using in the last 20 years or so and the reproducibility of the experimental results.

### 4.2. Growth Rate Enhancement of NCD Films with Different Amount of O_2_ and N_2_ Addition

We briefly discuss the growth rate of new NCD samples produced here. From Table 2 to Table 4, one can see that using different amount of N_2_ and O_2_ additives, the average growth rate of new NCD samples produced here was in the range from 2.1 μm/h to 6.7 μm/h, much higher than the typical growth rate of NCD films fabricated using Ar chemistry [14]. It seems that a much higher amount of oxygen than N_2_ addition did not benefit the growth rate enhancement more in the current study, However, from the value of growth rate, one cannot infer the quantitative contribution of each parameter on it unless all the other parameters are kept constant because the growth rate of CVD diamonds strongly depends on the overall growth conditions.

In addition, it has been shown that NCD films grown with nitrogen addition (from 0.6 up to 0.9 sccm) in CH_4_/H_2_ (from 7.5% to 15%) plasma grown at 3.5 kW and 20 kPa had a low coefficient of friction due to small surface roughness in comparison with MCD films grown with only CH_4_/H_2_ plasma using the same growth parameters [10]. Furthermore, it has been shown that nano-crystalline diamonds have great potential in tribology applications [38]. Therefore, exploitation of this new growth parameter range with N_2_ and O_2_ addition offers a simple way to tailor the growth of large area uniform NCD films with a high growth rate toward tribological applications such as wear-resistant and anti-fiction coatings upon the proper adaptation of standard high-power MPCVD conditions.

### 4.3. The Roles of a Small Amount of N_2_ and O_2_ Addition in Growth and Formation Mechanism of NCD Films under the Base Conditions Employed

Experimentally, the effects of a small fixed total amount of nitrogen and oxygen additives by changing their relative ratios on morphology, quality, and texture of diamond growth ranging from MCD to NCD by MPCVD have been clearly demonstrated [24]. Under conventional base conditions (for instance, that of D4 in this work), the critical role of an N_2_ additive on reducing grain sizes and eventually resulting in NCD formation has been illustrated with N_2_ addition only [39]. Regarding the formation of defects and incorporation of impurities in the growing diamond films, a question naturally rises. Does the use of nitrogen result in the diamond film doping? According to our experimental experiences and understanding of MPCVD diamond growth, in our case, the use of nitrogen in the gas phase with much a higher amount than the ppm level used in the related reference on a nitrogen-doped CVD single diamond [40] must result in diamond films with nitrogen doping. In fact, the nitrogen doping of diamonds grown by all CVD methods has been carried out by nitrogen addition or nitrogen-containing gas precursors into gas phase mixtures [7,8,19,40].

Under typical base conditions used for high-quality MCD growth, the key functions of a small amount of O_2_ addition on suppressing H impurity incorporation and improving quality of NCD films were illustrated when the total amount of N_2_ and O_2_ was fixed at 1 sccm [41] and by stabilizing a certain orientation or texture such as <100> orientation when an equal amount of N_2_ and O_2_ addition was employed [29] 

The main mechanisms of diamond growth from only CH_4_ and H_2_ precursor gases using MPCVD method can be found in a recent work on the two-dimensional modeling of diamond growth by MPCVD [42]. In that work, two-dimensional modelling of microwave plasma of CH_4_/H_2_ gas mixtures at power (1–3 kW) disclosed the radial and axial gradient distribution of absorbed power density in an MPCVD system [42]. This radial and axial gradient distribution of absorbed power density certainly will influence the distribution of growth species (for example H atom and CHx (x = 1,2,3) radicals) and substrate temperature, which are very important for CVD diamond growth. In our high-power 5 kW MPCVD system, we anticipate that the main growth mechanism remains the same as 4% CH_4_ dominated in H_2_ plasma. 

On the other hand, in present case, the addition of a small amount of N_2_ and O_2_ into the gas phase may result in the creation of more minor species such as CN, CO, N, O, NO, NH, and OH, which are also participating in the diamond growing process and competing with the major growth species, namely CHx (x = 1,2,3) radicals [43,44,45], and thus can drastically influence diamond growth. However, so far, we could not answer the following questions: how are these minor species involved in the growth process, and why MCD grows instead of NCD when the amount of O_2_ exceeds that of N_2_ up to a critical point? Therefore, the optimization of growth conditions for diamond growth by MPCVD systems is still needed, and more studies are ongoing to clarify the specific roles of N_2_ and O_2_ addition in the growth of NCD films by MPCVD.

## 5. Conclusions

In this work, a new operating parameter range for tailored fast growth of various NCD films by MPCVD was designed and established by fine-tuning the gas-phase compositions under base conditions conventionally used for MCD deposition. The highest average growth rate was 6.7 μm/h, achieved for the NCD film fabricated with equal amounts of N_2_ and O_2_ additives under the novel conditions employed here. When microwave power was fixed, the surface temperature of Si substrates was adjusted by two ways: (i) using Si wafers of different thickness, and (ii) using two types of distinct Mo substrate holder to ensure sole temperature effect on NCD growth under the conditions investigated. By these ways, significant difference between surface temperature of Si substrates was obtained and inferred by simulation, while uniform NCD films could still be formed on 2-inch Si wafers under otherwise identical growth conditions. Furthermore, the present results can provide a guideline for future design of experiments for the optimization of growth parameters for NCD synthesis by tuning the amounts and relative ratios of N_2_ and O_2_ additives into standard CH_4_/H_2_ gas phase chemistry. This particularly holds for MPCVD runs using typical parameters for growing large-grained polycrystalline diamond films. This may open a way to tailor the rapid growth of NCD films for mechanical applications such as in wear-resistant low-friction coatings.

## Figures and Tables

**Table 1:** Summary of both the literature and present work on using different quantity of N_2_ and O_2_ additives (flow unit: sccm) for NCD formation by high-power MPCVD.

N_2_ Addition Only		Both N_2_ and O_2_ Addition					
		Q_N2_ > Q_O2_		Q_N2_ = Q_O2_		Q_N2_ < Q_O2_	
1.0, 1.6	Ref. [19]	0.8/0.2	Ref. [24]	0.5/0.5	Ref. [24]	0.3/0.7	Ref. [25]
0.6–0.9	Ref. [10]	air	Refs. [11,26,27]	1.0/1.0	This work	1.0/3.0	This work

**Table 2:** Growth conditions for the first series of samples deposited on small silicon slices centered on the type 1 Mo holder together with some characteristics of the diamond samples for comparison.

Sample Name	Power (kW)	N_2_ (sccm)	O_2_ (sccm)	Holder Temp. (°C)	Growth Time (h)	Diamond Type	G. Rate (μm/h)
DNO1	3.0	0.5	0.5	309	29.7	NCD	2.6
DNO2	3.5	1.0	1.0	352	6.3	NCD	6.7
DN3	3.5	1.6	0	349	5.6	NCD	7.1
D4	3.2	0	0	290	10.5	MCD	2.5

**Table 3:** Thickness of Si substrates and some growth parameters of the second set of samples deposited at power 3.5 kW, with 1 sccm N_2_ and 1 sccm O_2_ after 2 h of deposition together with the RMS surface roughness denoted as Sq for comparison.

Sample	Thickness of Si (mm)	Holder Temp. (°C)	Growth Rate (μm/h)	RMS Sq (nm)
DNO5	3.0	349	4.2	327
DNO6	1.0	352	3.5	397
DNO7	5.0	347	3.8	291

**Table 4:** Growth condition for the third series of samples deposited with 1.0 sccm N_2_ and 3.0 sccm O_2_ at microwave power 3.2 kW and RMS Sq surface roughness.

Sample	Holder Type	Holder Temp. (°C)	Growth Time	Growth Rate (μm/h)	RMS Sq (nm)
DNO8	1	329	40 (min)	2.1	306
DNO9	1	327	10 + 10 (h)	2.1	524
DNO10	2	300	12 + 8 (h)	3.8	466

**Table 5:** List of the types of diamond products (including deduced ones) based on the amount of N_2_ and O_2_ addition when O_2_ ≥ N_2_ (flow unit: sccm) using microwave power between 3.0 and 3.5 kW and 4% CH_4_/H_2_ mixtures.

N_2_:O_2_	N_2_	O_2_	Product	Reference
**1:1**	1.0	1.0	NCD	This work
**1:2**	1.0	2.0	NCD	Deduced
**3:7**	0.3	0.7	NCD	Ref. [25]
**1:3**	1.0	3.0	NCD	This work
**1:4**	**0.2**	**0.8**	**MCD**	Ref. [24]

## Data Availability

Data are available in the manuscript.
